# Correlation between Negative Rapid Influenza Diagnostic Test and Severe Disease in Hospitalized Adults with Laboratory-Confirmed Influenza Virus Infection

**DOI:** 10.4269/ajtmh.19-0444

**Published:** 2020-08-31

**Authors:** Po-Yen Huang, Chia-ping Su, Shi-Wei Liu, Kuo-Chin Kao, Yu-Chia Hsieh, Ching-Tai Huang

**Affiliations:** 1 Division of Infectious Diseases, Department of Internal Medicine, Chang-Gung Memorial Hospital, Chang-Gung University College of Medicine, Taoyuan, Taiwan;; 2 Taiwan Centers for Disease Control, Ministry of Health and Welfare, Taipei, Taiwan;; 3 Institute of Epidemiology and Preventive Medicine, College of Public Health, National Taiwan University, Taipei, Taiwan;; 4 Department of Thoracic Medicine, Chang Gung Memorial Hospital, Taoyuan, Taiwan;; 5 Department of Respiratory Therapy, Chang Gung Memorial Hospital, Taoyuan, Taiwan;; 6 Division of Pediatric Infectious Diseases, Department of Pediatrics, Chang Gung Children’s Hospital, Chang Gung University College of Medicine, Taoyuan, Taiwan

## Abstract

False-negative rapid influenza diagnostic test (RIDT) results could mislead physicians to exclude an influenza diagnosis. We sought to evaluate the association between negative RIDT and intensive care unit (ICU) admission. We reviewed data from hospitalized adults with laboratory-confirmed influenza virus infections in a tertiary referral hospital in Taiwan from July 2009 to February 2011. The diagnosis was documented by real-time PCR or virus culture. Of 134 hospitalized adults infected with influenza virus, 38 (28%) were admitted to the ICU. Compared with RIDT-positive patients, the percentage of ICU admission was significantly higher among RIDT-negative patients (46% versus 13%, *P* < 0.001). The RIDT-negative patients had higher percentages of lower respiratory symptoms and more chest radiograph infiltrates. The time interval between the RIDT and antiviral treatment was longer in RIDT-negative than RIDT-positive patients (1.94 days versus 0.03 days, *P* < 0.001). Among patients presenting with mild illness, only a negative RIDT and delayed antiviral treatment were associated with ICU admission after adjusting for potential confounding factors. To conclude, patients with a negative RIDT were more likely to have severe disease and a delay in initiating antiviral treatment. Our findings should help improve treatment outcomes of hospitalized patients with influenza infection.

## INTRODUCTION

The influenza virus causes seasonal epidemics globally, with significant morbidity and mortality.^1,2^ Novel influenza may cause outbreaks without warning in any place of the world.^3^ Awareness is a prerequisite for diagnosis and treatment of this infectious disease that has great public health importance. Although most of the infected only suffer from mild illness, there is a population of patients with severe disease. Patients with severe influenza should get timely antiviral medication as previous observational studies have suggested lower mortality with early diagnosis and antiviral treatment in hospitalized patients.^4–8^

The rapid influenza diagnostic test (RIDT) is popular in point-of-care settings as it provides physicians results in a timely fashion. The test is easy to perform and provides results within 30 minutes. A positive test with its high specificity confirms the diagnosis. However, the sensitivity of RIDTs is limited, ranging from 50% to 70% across different clinical settings, including those of critically ill patients.^9–11^ A negative test with its low sensitivity often misleads physicians to exclude an influenza diagnosis.^12–14^ The false readings are especially problematic for patients with severe disease, as the chance for timely management with antiviral medication is often missed.

We studied RIDT results among our hospitalized adults with laboratory-confirmed influenza virus infection. We aimed to evaluate 1) whether a negative RIDT is associated with severity of illness as well as lower respiratory tract symptoms and 2) whether a negative RIDT causes a delay in initiating antiviral treatment.

## MATERIALS AND METHODS

### Settings and definitions.

**Figure 1.**
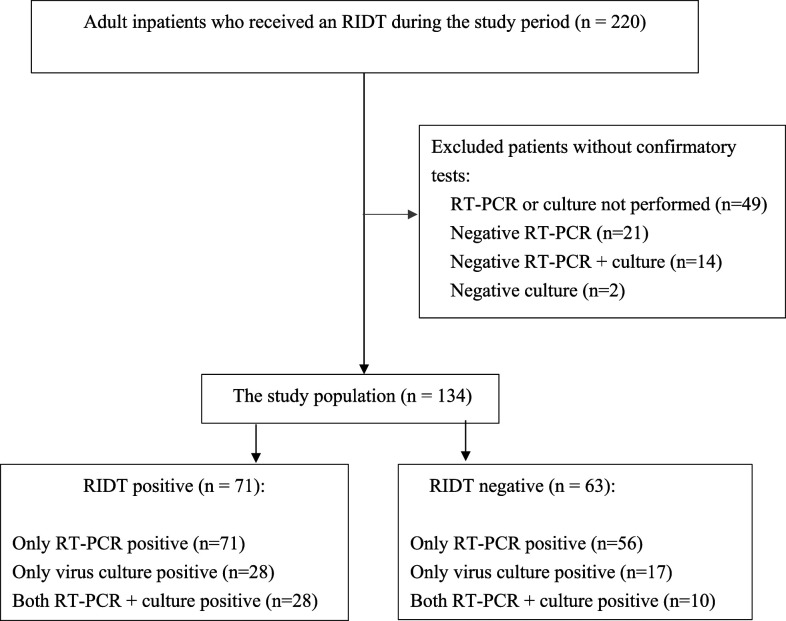
Flowchart of the study population selection from hospitalized adults with laboratory-confirmed influenza virus infections in a tertiary referral hospital, Taiwan, July 2009–February 2011.

This study was a retrospective analysis of data from a university-affiliated 3,900-bed tertiary referral hospital in Northern Taiwan with approximately 180,000 admissions annually. We reviewed data from the laboratory department of the hospital and identified all hospitalized adults (aged ≥ 18 years) who underwent RIDTs on presentation to the hospital from July 2009 to February 2011. Physicians performed RIDTs for patients who presented to the hospital with acute respiratory symptoms at their discretions. Those patients who did not undergo confirmatory tests or had negative real-time PCR (RT-PCR) test were excluded from the analysis (Figure 1). This study was approved by the institutional review board of the hospital (approval number: 100-1930B), and individual informed consent was waived because of its retrospective nature.

We reviewed medical records for demographic data, signs and symptoms at presentation, and the presence of comorbidities. Symptoms reviewed included fever, cough, sore throat, malaise, myalgia, rhinorrhea, dyspnea, hemoptysis, chest pain/tightness, nausea, vomiting, and diarrhea. The date of symptom onset was determined by chart review. Comorbidities included heart disease (defined as congestive heart failure, ischemic or valvular heart diseases, or hypertension), malignancy (hematologic malignancy or solid organ tumor), chronic obstructive lung diseases, kidney disease (creatinine level > 2.0 mg/dL or under hemodialysis), liver disease (total bilirubin level > 2.5 mg/dL or cirrhosis), immunosuppressant use (administration of steroids or immunosuppressant agents within 14 days before admission), and previous organ transplantation. Influenza-like illness was defined as fever (temperature of ≥ 37.8°C [≥ 100°F]) and cough or sore throat not attributable to another etiology, in accordance with the definition from the CDC in the United States.^15^

Chest radiograph findings were classified into three categories according to the criteria of Clinical Pulmonary Infection Score: normal, diffused infiltrates, or localized patches.^16^ Leukocytosis was defined as a leukocyte count > 12,000 cells/µL. Leukopenia was defined as a leukocyte count < 4,000 cells/µL. Thrombocytopenia was defined as a platelet count < 100,000 platelets/µL. Disease severity at presentation was evaluated by sepsis-related organ failure assessment (SOFA) score at the time of RIDT.^17^ A score of 0 was given to variables with missing information. We dichotomized these patients into mild illness (SOFA score 0–4) versus severe illness (SOFA score ≥ 5) at presentation.

The dates of symptom onset and the RIDT results were recorded. Timely antiviral treatment was defined as oseltamivir use within 2 days of symptom onset. The primary outcome in our study was intensive care unit (ICU) admission. In the hospital, the patient was admitted to the ICU because of the following conditions: 1) severe hypoxemia with PaO_2_/FiO_2_ < 100 mmHg, 2) respiratory failure requiring mechanical ventilation or extracorporeal membrane oxygenation, and 3) impending respiratory failure or clinical conditions requiring critical care.

### Specimen collection and laboratory testing.

Respiratory specimens were collected by physicians using throat swab or nasopharyngeal swab in clinical settings, sent to the laboratory within 30 minutes of sampling, and processed in the laboratory by trained technicians according to manufacturers’ instructions. All patients enrolled in this study were tested with the QuickVue influenza A + B test (Quindal, San Diego, CA), a commercially available lateral-flow immunoassay for the detection of influenza A and B virus antigens. For RT-PCR, viral RNA was extracted by MagNA PURE Autoextractor with MagNA Pure LC Total Nucleic Acid Isolation Kit (Roche Diagnostics, Mannheim, Germany). Extracted nucleic acid was amplified by an ABI 7000/7900 instrument with a commercial kit, TaqMan one-step RT-PCR mix reagent (Applied Biosystems, Foster City, CA). Specimens presumed to be positive for influenza A (cycle threshold ≤ 40) were then subtyped for A (H1N1) pdm09 and seasonal influenza A (H3N2). Virus culture also was performed by inoculation of the specimens into Madin-Darby Canine Kidney cells. Detailed methods have been previously described.^18^

### Statistical analysis.

Continuous variables were compared using Student’s *t*-test or the Mann–Whitney *U*-test depending on the validity of the normal distribution assumption. Binomial variables were compared using χ^2^ test or Fisher’s exact test, wherever appropriate. A two-tailed *P*-value < 0.05 was considered statistically significant in univariate analysis. A logistic regression model was used in multivariate analysis to determine the independent predictor(s) for ICU admission. Because of limited case number of the study and collinearity between several variables, the factors were entered into the model when they had a *P*-value < 0.20 and were with clinical significance. All statistical analyses were performed using R statistical software (R version 3.3.0, R Foundation for Statistical Computing, Vienna, Austria).

## RESULTS

During the study period, we identified 134 RT-PCR or culture-confirmed influenza virus–infected inpatients who underwent RIDTs at presentation (Figure 1). Of these patients, 71 (53%) were RIDT positive. There were no differences with respect to age, gender, or comorbidities, except that RIDT-positive patients were more likely using immune suppressants (13% versus 3%, *P =* 0.046) (Table 1). Regarding symptoms and signs on presentation, RIDT-positive patients were more likely to have upper respiratory tract symptoms such as sore throat and nasal symptoms, whereas RIDT-negative patients mainly presented with lower respiratory tract symptoms such as dyspnea and hemoptysis. Rapid influenza diagnostic test–negative patients had a higher percentage of localized infiltrates on chest radiograph, a higher C-reactive protein level, and a higher SOFA score (Table 1). Virus subtypes were identified by RT-PCR in 122 of 134 patients. Approximately two-thirds of the cases involved the A (H1N1) pdm09 virus, whereas one-third of cases were caused by the seasonal A (H3N2) virus (Table 1).

**Table 1:** Demographic data and clinical characteristics of hospitalized patients with positive and negative RIDT results

Variable	All (*n* = 134)	RIDT positive (*n* = 71)	RIDT negative (*n* = 63)	*P*-value
Demographic data				
Age (years)	51.6 ± 19.1	50.0 ± 19.0	53.2 ± 19.3	0.338
Gender, male	69 (52)	36 (51)	33 (52)	0.846
Comorbidities				
Heart disease	24 (18)	13 (18)	11 (17)	0.898
Malignancy	18 (13)	10 (14)	8 (13)	0.814
Chronic obstructive lung disease	19 (14)	11 (15)	8 (13)	0.643
Kidney disease	20 (15)	8 (11)	12 (19)	0.207
Diabetes mellitus	34 (25)	17 (24)	17 (27)	0.686
Liver disease	11 (8)	4 (6)	7 (11)	0.249
Immunosuppressant use	11 (8)	9 (13)	2 (3)	*0.046*
Previous organ transplantation	3 (2)	3 (4)	0 (0)	0.247
ILI contact or cluster	19 (14)	13 (18)	6 (10)	0.146
Symptoms and signs on presentation				
ILI	118 (88)	63 (89)	55 (87)	0.799
Fever	125 (93)	66 (93)	59 (94)	1.000
Cough	127 (95)	68 (96)	59 (94)	0.706
Sore throat	67 (50)	43 (61)	24 (38)	*0.009*
Malaise/myalgia	82 (61)	49 (69)	33 (52)	*0.049*
Nasal symptoms	48 (36)	30 (42)	18 (29)	0.099
Subjective dyspnea	65 (49)	28 (39)	37 (59)	*0.026*
Hemoptysis	12 (9)	3 (4)	9 (14)	*0.042*
Chest pain/tightness	19 (14	11 (16)	8 (13)	0.643
Headache	27 (20)	15 (21)	12 (19)	0.765
Nausea/vomiting	24 (18)	11 (16)	13 (21)	0.438
Diarrhea	14 (10)	7 (10)	7 (11)	0.813
Chest radiograph and laboratory data on presentation				
Localized infiltrates on chest radiograph	48 (36)	17 (24)	31 (49)	*0.002*
Leukocytosis or leukopenia	34 (25)	15 (21)	19 (30)	0.230
Thrombocytopenia	13 (10)	6 (8)	7 (11)	0.604
C-reactive protein (mg/L)	86.0 ± 77.3	65.8 ± 64.3	105.4 ± 84.1	*0.006*
Virus type				
A (H1N1 pdm09)	81 (60)	47 (66)	34 (54)	0.839
A (H3N2)	41 (31)	23 (32)	18 (29)	–
A (undetermined)	12 (8)	1 (1)	11 (17)	–

ILI = influenza-like illness; RIDT = rapid influenza diagnostic test. Categorical data are presented as no. (%) of subject, and continuous data are expressed as mean ± SD unless otherwise indicates. An italic *P*-value indicating statistically significant and *P* < 0.05.

Compared with RIDT-positive patients, the percentage of severe illness (SOFA score ≥ 5) on admission was significantly higher among RIDT-negative patients (32% versus 15%, *P* = 0.026) (Table 2). After symptom onset, the RIDT-negative patients were admitted to the hospital later than the RIDT-positive patients (3.11 ± 3.05 versus 1.27 ± 1.68 days, *P <* 0.001). As a consequence, the time interval between symptom onset and RIDTs was longer as well (4.30 ± 3.00 versus 1.83 ± 1.54 days, *P* < 0.001). In addition, RIDT-negative patients were also less likely to receive oseltamivir treatment than RIDT-positive patients (79% versus 97%, *P <* 0.001). Even when oseltamivir was prescribed in these negative patients, the duration between RIDTs and prescription was longer (1.94 ± 2.41 days versus 0.03 ± 0.17 days, *P <* 0.001). Compared with RIDT-positive patients, RIDT-negative patients had longer duration between symptom onset and the prescription (6.24 ± 4.03 days versus 1.88 ± 1.58 days, *P* < 0.001). Overall, timely administration of oseltamivir was significantly less possible for RIDT-negative than RIDT-positive patients (10% versus 73%, *P* < 0.001) (Table 2). Nearly half of the RIDT-negative patients were admitted to the ICU, which was significantly higher than the RIDT-positive patients (46% versus 13%, *P* < 0.001). The difference in in-hospital mortality between RIDT-negative and RIDT-positive patients was not statistically significant (16% versus 8%, *P* = 0.186).

**Table 2:** Impact of RIDT among hospitalized patients

Variable	All (*n* = 134)	RIDT positive (*n* = 71)	RIDT negative (*n* = 63)	*P*-value
Disease severity at presentation				
SOFA score	2.6 ± 3.2	1.9 ± 3.0	3.4 ± 3.3	*0.006*
Mild illness (SOFA score ≤ 4)	103 (77)	60 (85)	43 (68)	–
Severe illness (SOFA score ≥ 5)	31 (23)	11 (15)	20 (32)	*0.026*
Antiviral treatment and RIDT				
Oseltamivir use	119 (89)	69 (97)	50 (79)	*0.001*
Timely oseltamivir use*	58 (43)	52 (73)	6 (10)	*< 0.001*
Symptom onset to admission (days)	2.1 ± 2.6	1.3 ± 1.7	3.1 ± 3.1	*< 0.001*
Symptom onset to RIDT (days)	3.0 ± 2.6	1.8 ± 1.5	4.3 ± 3.0	*< 0.001*
Admission to oseltamivir use (days)	1.6 ± 2.6	0.6 ± 1.7	2.9 ± 3.0	*< 0.001*
RIDT to oseltamivir use (days)	0.8 ± 1.8	0.0 ± 0.2	1.9 ± 2.4	*< 0.001*
RIDT to real-time PCR/culture	0.9 ± 1.1	0.8 ± 1.0	1.1 ± 1.2	0.123
Outcome				
Intensive care unit admission	38 (28)	9 (13)	29 (46)	*< 0.001*
Mechanical ventilation	31 (23)	9 (12)	22 (35)	*0.002*
Extracorporeal membrane oxygenation	7 (5)	2 (3)	5 (8)	0.253
30-day mortality	14 (10)	6 (8)	8 (13)	0.422
In-hospital mortality	16 (12)	6 (8)	10 (16)	0.186

RIDT = rapid influenza diagnostic test. Categorical data are presented as no. (%) of subject, and continuous data are expressed as mean ± SD unless otherwise indicates. An italic *P*-value indicating statistically significant and *P* < 0.05.

* Defined as initiation of oseltamivir within 48 hours of symptom onset.

Multivariate analysis revealed that negative RIDT results remained independently associated with ICU admission (adjusted odds ratio [OR]: 22.63; 95% CI: 1.61–592.31; *P* = 0.035), after controlling for comorbid illness, disease severity, virus types, and antiviral treatment (Table 3). Among patients with mild illness on presentation (SOFA score ≤ 4), the odds of admission to the ICU for RIDT-negative patients were greater than those of the RIDT-positive patients (crude OR: 48.02; 95% CI: 2.75–837.86; *P* < 0.001). The chance for admission to an ICU significantly increased for patients with delayed antiviral treatment compared with those with timely medication (crude OR: 27.87; 95% CI: 1.60–484.86; *P* < 0.001) (Table 4). However, the RIDT result or the antiviral treatment was not associated with ICU admission among patients with severe illness on presentation (SOFA score ≥ 5). The virus type was not associated with ICU admission among patients of either mild or severe illness (Table 4).

**Table 3:** Factors associated with ICU admission among hospitalized patients with laboratory-confirmed influenza

Variable	ICU admission (*n* = 38)	Non-ICU admission (*n* = 96)	Univariate	Multivariate*	
			*P*-value	Odds ratio (95% CI)	*P*-value
Demographic data					
Age (years)	50.4 ± 16.5	52.2 ± 20.2	0.634	–	–
Gender, male	25 (66)	44 (46)	0.037	1.90 (3.70–11.37)	0.449
Comorbidities					
Heart disease	5 (13)	19 (20)	0.367	–	–
Malignancy	3 (8)	15 (10)	0.237	–	–
Chronic obstructive lung disease	4 (11)	15 (16)	0.446	–	–
Kidney disease	13 (34)	7 (7)	*< 0.001*	0.72 (0.04–13.04)	0.820
Diabetes mellitus	10 (26)	24 (25)	0.875	–	–
Liver disease	7 (18)	4 (4)	*0.012*	9.38 (0.26–635.47)	0.274
Immunosuppressant use	1 (3)	10 (10)	0.179	0.098 (0.003–1.51)	0.117
Previous organ transplantation	1 (3)	2 (2)	> 0.999	–	–
Influenza-like illness Contact or cluster	5 (13)	14 (15)	0.831	–	–
Symptoms and signs on presentation					
Influenza-like illness	4 (11)	12 (13)	> 0.999	–	–
Fever	35 (92)	90 (94)	0.713	–	–
Cough	37 (97)	90 (94)	0.673	–	–
Sore throat	11 (29)	56 (58)	*0.002*	–	–
Malaise/myalgia	17 (45)	65 (68)	*0.014*	–	–
Nasal symptoms	8 (21)	40 (42)	*0.025*	–	–
Subjective dyspnea	36 (95)	38 (30)	*< 0.001*	–	–
Hemoptysis	9 (24)	3 (3)	*< 0.001*	–	–
Chest pain/tightness	5 (13)	14 (15)	0.831	–	–
Headache	4 (11)	23 (24)	0.081	–	–
Nausea/vomiting	8 (21)	16 (17)	0.551	–	–
Diarrhea	8 (21)	6 (6)	*0.024*	–	–
Chest radiograph and laboratory data on presentation					
Localized infiltrates on chest radiograph	35 (92)	13 (14)	*< 0.001*	70.86 (10.93–967.08)	*< 0.001*
Leukocytosis or leukopenia	15 (39)	19 (20)	*0.018*	–	–
Thrombocytopenia	5 (13)	8 (8)	0.517	–	–
C-reactive protein (mg/L)	135.4 ± 91.0	61.0 ± 44.8	*< 0.001*	–	–
Virus type					
A (H1N1 pdm09)	30 (79)	51 (53)	*0.015*	–	–
A (H3N2)	5 (13)	36 (38)	–	0.48 (0.041–4.31)	0.521
A (undetermined)	3 (8)	9 (25)	–	0.34 (0.017–6.60)	0.467
Disease severity at presentation					
SOFA score	6.4 ± 3.2	1.1 ± 1.5	*< 0.001*	–	–
Severe illness (SOFA score ≥ 5)	26 (68)	5 (5)	*< 0.001*	62.94 (5.39–1934.42)	*0.005*
Antiviral treatment and RIDT					
Timely oseltamivir use†	8 (21)	50 (52)	*0.001*	1.20 (0.088–15.82)	0.886
Symptom onset to admission (days)	3.8 ± 3.5	1.5 ± 1.7	*< 0.001*	–	–
Symptom onset to RIDT (days)	4.5 ± 3.5	2.4 ± 1.9	*< 0.001*	–	–
Admission to oseltamivir use (days)	1.9 ± 2.7	1.4 ± 2.6	0.363	–	–
RIDT to oseltamivir use (days)	1.3 ± 2.5	0.7 ± 1.4	0.186	–	–
RIDT to real-time PCR/culture	1.1 ± 1.5	0.8 ± 0.9	0.185	–	–
Negative RIDT result	29 (76)	34 (35)	*< 0.001*	22.63 (1.61–592.31)	*0.035*

RIDT = rapid influenza diagnostic test; SOFA score = sepsis-related organ failure assessment score. Categorical data are presented as no. (%) of subject, and continuous data are expressed as mean ± SD unless otherwise indicates. An italic *P*-value indicating statistically significant and *P* < 0.05.

* The factors included in the multivariate model were gender, kidney disease, liver disease, immunosuppressant use, pulmonary infiltrates, SOFA score ≥ 5, virus type (H3N2 vs. H1N1 pdm09), timely oseltamivir use, and negative RIDT result. Other potential factors were not included because of limited case number and multicollinearity.

† Defined as initiation of oseltamivir within 48 hours of symptom onset.

**Table 4:** Factors associated with intensive care unit admission for different disease severity on presentation to the hospital

	Mild illness (SOFA score ≤ 4) (*n* = 103)		Severe illness (SOFA score ≥ 5) (*n* = 31)	
	Crude OR (95% CI)	*P*-value	Crude OR (95% CI)	*P*-value
RIDT results				
Positive	1	–	1	–
Negative	48.02 (2.75–837.86)	*< 0.001*	1.26 (0.18–8.97)	> 0.999
Virus type				
A (H1N1) pdm09	1	–	1	–
A (H3N2)	0.15 (0.02–1.26)	0.082	0.57 (0.05–6.98)	0.553
Antiviral treatment				
Timely*	1	–	1	–
Delayed	27.87 (1.60–484.86)	*< 0.001*	1.50 (0.21–10.79)	> 0.999

RIDT = rapid influenza diagnostic test; SOFA score = sepsis-related organ failure assessment score. An italic *P*-value indicating statistically significant and *P* < 0.05.

* Defined as initiation of oseltamivir within 48 hours of symptom onset.

## DISCUSSION

In this retrospective study of hospitalized adults with influenza virus infection, we demonstrated the correlation between a negative RIDT and severe influenza. Rapid influenza diagnostic test–negative patients displayed pronounced lower respiratory symptoms and signs, and laboratory profiles of greater severity. Intensive care unit admission was more common among RIDT-negative patients. A negative RIDT result was independently associated with ICU admission after adjusting for comorbidity, disease severity, virus types, and antiviral treatment.

Nonspecific clinical presentations make influenza diagnosis a challenge for the clinicians. Rapid influenza diagnostic test is an easy test to perform with results available in less than 30 minutes. Physicians tend to perform the RIDT as confirmation for a diagnosis. However, the sensitivity of the RIDT is limited when compared with that of other confirmatory laboratory tests.^19,20^ Beyond poor sensitivity, a study demonstrated that the severity of influenza did not correlate with the viral load in the upper respiratory tract.^21^ As RIDT positivity is proportional to viral load in the pharyngeal samples, severe influenza may present with a negative RIDT.^18,22,23^ In addition, it has been shown that RIDT-negative cases were significantly overrepresented among severe influenza.^12–14^ Our findings further corroborated the correlation between negative RIDT and severe influenza. This correlation was more apparent among patients with mild disease (SOFA score ≤ 4) than the others (SOFA score ≥ 5) on presentation. Therefore, clinicians have to interpret a negative RIDT with caution. In patients with influenza who need hospitalization, a negative RIDT implies the strong possibility for severe disease and a poor outcome requiring admission to the ICU.

Viral loads detected in the upper respiratory tract decrease with time in patients with influenza A infection. This pattern of viral shedding is the natural course of recovery from the disease in most of the patients.^24^ In our specific population of influenza patients who needed to be hospitalized, a negative RIDT meant that the viral load in their upper respiratory tract had decreased as well. Compared with the RIDT-positive cases, these RIDT-negative patients had longer symptom onset to hospital presentation and poorer outcome. One plausible hypothesis is that the infection progresses from the upper respiratory tract to the lower respiratory tract. As a result, these patients had lower respiratory tract involvement, as well as laboratory data profiles of systemic inflammatory responses. Reddy et al.^25^ have examined the differences in influenza diagnostic yields from upper and lower respiratory tract specimens and observed worse clinical outcomes in upper respiratory tract negative versus upper respiratory tract positive subjects. However, future study may prove this hypothesis by examining the viral loads of both lower and upper respiratory tracts.

Oseltamivir is the antiviral medication for influenza virus infection, although the effectiveness to prevent severe illness is controversial.^26^ Some observational studies have demonstrated that early oseltamivir administration was associated with fewer complications and favorable outcomes among hospitalized or critically ill patients.^26,27^ Studies have also shown that delayed oseltamivir use was associated with severe illness in influenza virus infection.^28–30^ In our analysis, the administration of oseltamivir was significantly delayed after the hospital presentation as well as the symptom onset among RIDT-negative patients when compared with RIDT-positive patients. Also, delayed oseltamivir treatment was associated with ICU admission among patients with mild disease (SOFA score ≤ 4) on presentation. The cause–effect between delayed antiviral medication and ICU admission cannot be documented among those with severe disease (SOFA score ≥ 5). However, the finding that clinicians hesitated to initiate antiviral treatment for negative RIDT patients is problematic as the potential benefit of timely oseltamivir for prevention of severe influenza cannot be overlooked.^27,31^ Our findings during the study period of 2009–2011 have changed our practices and we have since advocated for antiviral treatment of patients hospitalized because of respiratory tract infections, especially during influenza seasons, even if the RIDT is negative. This practice coincides with the current recommendations from literature with relatively weak evidence.^32,33^

The A (H1N1) pdm09 virus was the prevalent subtype in our study. Nevertheless, it was not the only strain present among our cases. One-third of the patients were infected with seasonal A (H3N2), a subtype that co-circulated with A (H1N1) pdm09 at a smaller magnitude during the study period in Taiwan.^34^ Although A (H1N1) pdm09 virus infection was believed to be more likely to progress to critical illness, seasonal A (H3N2) was equally important in our study after stratification by disease severity. This finding is supported by a previous study reporting that both A (H1N1) pdm09 and seasonal A (H3N2) can cause severe illness.^35^ Vigorous studies prompted detection of influenza virus in these patients during the pandemic of A (H1N1) pdm09 in 2009. Currently, it is still possible that the real number of hospitalized patients with influenza virus infection was underestimated. As influenza is a potentially treatable disease, we propose diagnostic studies of influenza virus infection for all patients with severe lower respiratory tract illnesses of undetermined etiology.

## LIMITATIONS

Our findings are subject to limitations because of the retrospective nature. First, RIDT-positive patients may have been admitted to the hospital with less severity of illness with the panic at the beginning of the 2009 pandemic. Unnecessary admission may exaggerate the disparity between RIDT-negative and RIDT-positive populations. Second, on uncertainty about the diagnosis, physicians tended to perform RT-PCR and virus culture for critically ill patients who had negative RIDT as the confirmation. The study pool of our patients with documented influenza virus infection with RT-PCR and virus culture may have included more patients with critical illness and negative RIDT. Third, we excluded the patients who did not undergo confirmatory test, and this could bias the results of our study. There were 49 RIDT-positive patients who did not have confirmatory test performed. One of them was critically ill and admitted to the ICU. Moreover, this study excluded those RIDT-negative patients who may truly have mild influenza infection and did not undergo confirmatory testing. Fourth, we do not have complete information on coinfection. Because the importance of coinfection is mainly for the disease of later stage, our results should include the impact of coinfection or subsequent superinfection. Finally, although this study spanned over 2 years, it was limited to the cases of one medical facility. During the study period across two influenza seasons, there could be differences in testing practices, clinical presentations, or frequency of the primary outcome. Despite these limitations, a negative RIDT in laboratory-confirmed influenza A virus infection was associated with an almost 50% chance for progression to critical illness and ICU admission. Our findings have implications for the diagnosis and treatment of hospitalized patients with influenza infection. As RIDT-negative patients are likely to become critically ill during influenza seasons, physicians should assess the patient and initiate appropriate antiviral therapy in time, regardless of the RIDT results.

## CONCLUSION

In conclusion, negative RIDT results were independently associated with delayed antiviral treatment and ICU admission among our adult patients hospitalized with laboratory-confirmed influenza virus infection during this study period. Interpretation of RIDT results in the clinical setting has to be performed under caution. A negative RIDT cannot exclude influenza virus infection and could be a warning sign of progression to severe disease. A subsequent delay in antiviral medications may adversely impact the clinical outcome. Antiviral therapy may be started when the RIDT is negative and influenza is prevalent in the community.
